# Epidemiology of injuries presenting to the accident centre of Korle-Bu Teaching Hospital, Ghana

**DOI:** 10.1186/s12873-019-0252-3

**Published:** 2019-07-20

**Authors:** Paa-Kwesi Blankson, Joachim K. A. Amoako, Kwaku Asah-Opoku, Francis Odei-Ansong, Margaret Y. Lartey

**Affiliations:** 1 Department of Oral & Maxillofacial Surgery, Korle-Bu Teaching Hospital, Accra, Ghana; 2 Department of Surgery, Korle-Bu Teaching Hospital, Accra, Ghana; 3 School of Medicine and Dentistry, College of Health Sciences, University of Ghana, P.O. Box KB-20, Accra, Ghana; 4 Department of Orthopaedic Surgery, Korle-Bu Teaching Hospital, Accra, Ghana; 5 Department of Medicine and Therapeutics, Korle-Bu Teaching Hospital, Accra, Ghana

**Keywords:** Injury, Accident, Epidemiology, Aetiology, Ghana

## Abstract

**Background:**

Injuries directly lead to 5 million deaths every year, accounting for 9% of all deaths worldwide. While knowledge of the pattern of injuries is essential to plan health interventions to reduce the incidence of injuries, these are not thoroughly described in Ghana. The aim of this study was to describe the epidemiology of injuries seen at the Accident centre of the Korle-Bu Teaching Hospital, Ghana’s main referral hospital.

**Method:**

A retrospective review of two-year records of all patients who attended the Accident centre of the Korle-Bu Teaching Hospital from January 2016 to December 2017 was done. Data on the cause of injuries was the main focus of this review.

**Results:**

A total of 17,860 patients’ records were included in the study. There were 12,116 (67.8%) males and 5,744 (32.2%) females. The ages of the patients seen during the period ranged from three (3) days to 101 years. The overall mean age was 27.9 (±18.2). Majority of the injuries resulted from road traffic accidents and falls, accounting for 39.1 and 19.7% respectively.

**Conclusion:**

Road Traffic accidents (RTA), especially motorcycle related, are a significant cause of injuries in Ghana. Future studies should focus on interventions that can reduce the incidence of RTA’s to reduce the number of injuries that present to the Korle-Bu Teaching Hospital.

## Background

The social and economic impact of injuries cannot be overlooked. Every year, injuries caused by violence, road traffic accidents, falls, drowning, and burns, among others, result in over 5 million deaths in the world [1]. Its incidence is rising, and the United Nations has targeted its reduction as a major component of the 2030 Agenda for Sustainable Development Goals (SDG) [2]. Over the past century, there has been a change in the pattern of causes of death in the world, with the growing importance of road traffic accidents (RTA) in particular [3].

The World Health Organisation (WHO) has identified road injury as the tenth most frequent cause of death worldwide, and predicts a 40% increase in global deaths owing to injuries in general by 2030 (WHO, 2016). The burden of injuries is said to be decreasing in high-income countries while increasing in sub-Saharan Africa [4]. The prevention and management of injuries should, therefore, be of concern for the West Africa sub-region if it is to reduce morbidity and mortality.

The trauma system in Ghana is evolving, with the gradual development of an effective emergency medical system (EMS) and prehospital system. However, there is currently, a wide gap in access to timely prehospital care and EMS. Understanding the nature and distribution of injuries will provide baseline data and contribute significantly to the formulation and direction of national policies regarding injury management and injury prevention, in reducing its social and economic impact on countries such as Ghana. The aim of this retrospective study was to describe the epidemiology of injuries seen at a major referral centre in Accra, the capital of Ghana.

## Methods

### Study design, study site and participants

We reviewed the medical records of all patients who reported to the Accident centre of the Korle-Bu Teaching Hospital from January 2016 to December 2017, where the study took place.

The Korle Bu Teaching Hospital is the largest health facility, and premier teaching hospital in Ghana. With a bed capacity of over 2000, it serves as the main referral center for the entire southern Ghana and beyond. It also serves as a training center for medical doctors, nurses and other health professionals. The hospital has 17 clinical and diagnostic departments/units which includes the Accident and emergency department (housing the Accident center), where this study took place. It is the point of entry into the hospital for victims of road crashes, burns, bites, and foreign body ingestion. The centre attends to ‘walk-in’ and referred trauma patients of all ages, from primary care, primary hospitals and secondary hospitals in the southern part of Ghana and beyond. It, therefore, has a wide catchment area with an ill-defined population**.**

### Inclusion/exclusion criteria

Records for all patients with injuries seen at the Accident centre within the study period were included in the review. Injured patients were those who had sustained physical insult of sudden onset and severity which required immediate medical attention. These included road traffic crashes, industrial/occupational injuries, sports, assault/interpersonal violence, sexual assault, gunshot injury, play and fall injuries, dog bite, burns, foreign body, injury from seizure/sudden loss of consciousness, fallen object, stepped on a sharp object, door hitting finger, trip/collision, and intentional self-inflicted injuries. Patient records with inadequate information were excluded.

### Data collection, tools and variables

Data was collected from summary charts and available folders, supplemented with triage records. These were entered into a computerized abstraction table using Microsoft Excel 2010. Variables recorded were age, sex, date of presentation, cause of the injury, the site of the injury and outcome of emergency management. The International Classification of External Causes of Injury (ICECI) by WHO was used in the injury site classification system for this study [5]. The role of the patients involved in Road Traffic Accidents (RTA) was also recorded as being a pedestrian, bus passengers, or motorist (which included occupants of private cars). Patients who were seen/admitted at the Accident center were managed/stabilized, and discharged/transferred (if necessary) to another department for definitive care. The mortality rate was, therefore measured, for the period from admission to the time of discharge or transfer out of the department.

### Statistical analysis

Variables were analyzed using STATA (version 14). Background characteristics for all respondents were described, and descriptive summaries for all variables reported. Chi-square test was used to compare categorical variables with consequent test of association, assuming an alpha level of 0.05.

## Results

Eighteen thousand, one hundred and seventeen (18,117) patients with injuries were seen at the Accident centre of the Korle-Bu Teaching hospital within the study period. Of these, 17,860 were included in the study. The patients consisted of 12,116 (67.8%) males and 5,744 (32.2%) females. The ages ranged from three (3) days old to 101 years old. The overall mean age was 27.9 (±18.2). The mean age among the males was 27.6 (±16.7) years, while that among females was 28.5 (±21.1) years.

People in their third decade of life were the most injured in this study (3,447, 24.8%). Patients under 5 years comprised 12.4% (2,214) of the study population. While 17.8% percent of injured patients were brought in with an ambulance, most injured patients ‘walked-in’ to the department, and 15.9% were not ambulant but brought in by other means (carried). Most injured patients seen at the Emergency Department (68.7%) were managed and discharged from the Accident centre**.** However, 30.2% were transferred for primary management by other departments and 182 (1.0%) died (Table 1).

**Table 1:** Background characteristics

Variable	Number	Percent
Sex		
Male	12,116	67.8
Female	5,744	32.2
Age		
< 18	11,678	65.4
18–65	5,883	32.9
> 65	299	1.7
Referral		
Referred	2,322	13.0
Not referred	15,538	87.0
Mode of entry^a^		
Walk-in	11,820	66.3
Carried	2,840	15.9
Ambulance	3,175	17.8
Outcome		
Treated and Discharged	12,262	68.7
Admitted	5,416	30.3
Died	182	1.0

^a^ Documented in 17,835 patients

**Fig. 1:**
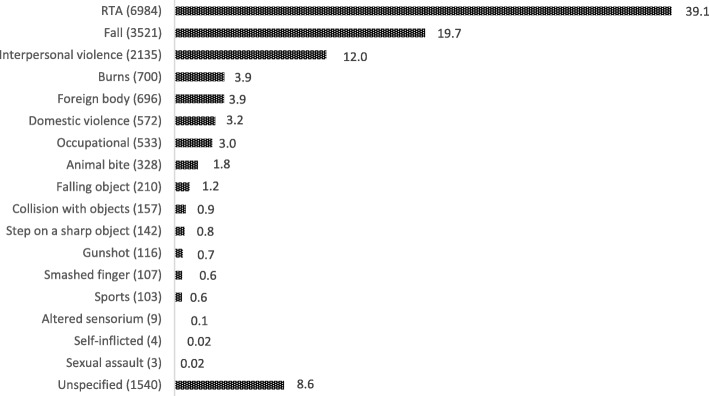
Pattern of the aetiology of injuries

Road traffic incidents accounted for most injuries presenting to the facility (39.1%). This was followed by injuries from falls, assault, and burns, which caused 19.7, 12 and 3.9% of injuries within the study period. The distribution of injuries from these three major aetiologies is shown in Table 2. Only three cases of injuries from sexual assault were reported to the department, while four and nine individuals were seen for self-inflicted injuries and sudden loss of consciousness respectively (Fig. 1).

**Table 2:** Distribution of injuries from RTA, Assault, and Falls

Variable	RTA (%)	Assault (%)	Fall (%)
Sex			
Male	4,884 (69.9)	1592 (74.5)	2151 (61.1)
Female	2,100 (30.1)	544 (25.5)	1370 (38.9)
Age			
< 18	4,594 (39.3)	1,210 (10.4)	2,560 (21.9)
18–65	2,306 (39.2)	915 (15.6)	814 (13.8)
> 65	84 (28.1)	11 (3.7)	147 (49.2)
Outcome			
Treated and discharged	4519 (64.7)	1703 (79.7)	2541 (72.2)
Admitted	2353 (33.7)	422 (19.8)	955 (27.1)
Died	112 (1.6)	11 (0.5)	25 (0.7)

Road Traffic accidents and falls accounted for 61.5 and 13.7% of deaths respectively, while interpersonal violence accounted for 6.0% of the deaths at the study site. Among the deaths caused by road crashes, 50.1% occurred in pedestrians, 31.2% in passengers and 18.7% occurred in motorists. The cause of death was not specified in 6.6% cases, and all other aetiologies accounted for the remaining 12.2% of deaths.

Forty-one percent (2,838), of the road traffic injuries involved passengers of commercial vehicles, 38% (2,668) involved pedestrians and 21% (1,478) involved motorists and occupants of privately owned vehicles.

Of all persons injured from road traffic crashes, 25.5% (1,779) were related to motorbike accidents. Four hundred and sixteen (23.4%) of these involved the main or pillion rider, while the remaining 1,363 (76.6%) involved pedestrians. Also, only 16.3% (68) of injured motor-bike riders (main or pillion) indicated having a helmet on at the time of injury.

**Fig. 2:**
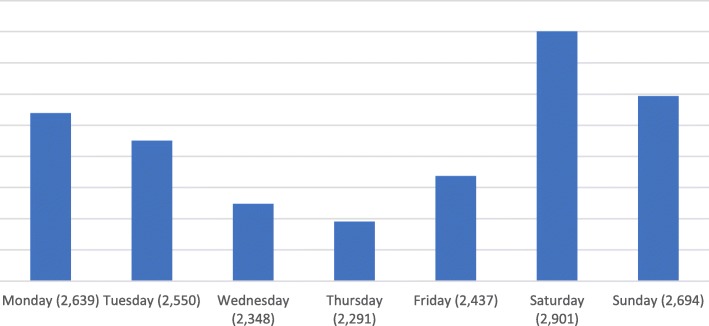
Total daily attendance due to injuries

**Fig. 3:**
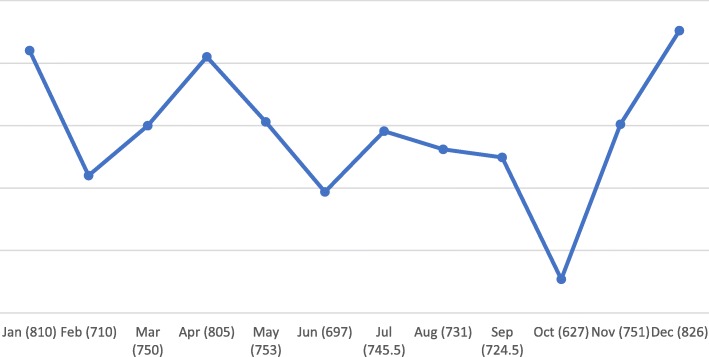
Mean monthly distribution of attendance due to injuries

There was a variable pattern of presentation across the days of the week and the months of the year. The highest number of injuries were recorded during weekends, peaking on Saturdays, with a cumulative attendance of 2, 901 (Fig. 2). December and January had the highest mean attendance followed by April for the two years of review (Fig. 3).

The head region was the most frequent site of injury accounting for 32.5% of all recorded injury sites and locations. This, made up of the neurocranium as well as the viscerocranium, included the face, jaws, ears, nose, and eyes. The least reported injury site was the genitals, accounting for 0.6% of all injuries (Table 3).

**Table 3:** Injuries by body region

Body region	Number	Percent (%)^a^
Head (Including face, jaws, ears, eyes & nose)	5803	32.5
Neck	638	3.6
Thorax	1729	9.7
Abdomen/Lower back/Pelvis	1713	9.6
Shoulder/Upper arms	1650	9.2
Elbow/Forearm	1048	5.9
Wrist and hand	2448	13.7
Hip/Thigh	869	4.9
Knee, lower leg	2921	16.4
Ankle/foot	1323	7.4
Genitals	107	0.6

^a^ Multiple sites of injuries included

## Discussion

This study set out to determine the pattern of injuries presenting to the accident centre of a major hospital in sub-Saharan Africa. In the two-year study, the majority of the burden of injuries was borne by road traffic accidents and falls, accounting for 39.1 and 19.7% respectively (Fig. 1). Although injuries are generally thought to be preventable, they account for a significant proportion of global mortality, a prevalence which requires international conscientious efforts to control. Road traffic injuries have taken a greater share of this burden in several regions over the past several decades due to the production of higher velocity vehicles, with likely increase in the prevalence as more people, especially in LMIC are gradually inclining to own vehicles, a phenomenon associated with economic growth [6]. The proportion of injuries attributable to RTAs in our study (39.1%) is consistent with findings of 43.9% in Tanzania [7] and 49% in Kampala, Uganda [8]. While this finding is consistent with many reports, even beyond Africa [9, 10], our study puts the associated morbidity, mortality, and cost of RTA in Ghana into perspective. We found that most of the patients injured through RTAs were passengers, and deaths resulting from RTA was mostly accounted for by pedestrians. Afukaar et al., in their review of road traffic injuries in Ghana using police data, likewise observed that most casualties of RTA were passengers followed by pedestrians, while the inverse was observed for RTA fatalities [11]. This finding, also corroborated by Hesse and Ofosu, (2014), therefore, suggests that pedestrians and bystanders are the categories of individuals at most risk of mortality in road traffic accidents. While seat-belts, helmets, and enforced blood alcohol limits lead the way in public health policies to reduce traffic injuries, roadside safety and proper use of the roads by pedestrians, sellers and non-traveling users of roads should also be given critical consideration.

Motorcycles have gradually become a popular means of travel in many places in sub-Saharan Africa, but quite unfortunately, not without grave consequences. The mortality and morbidity associated with the vehicle in LMICs have been well documented [12, 13], but is yet to see a full commitment by governments and policymakers. Even in high-income countries, it has been reported that the risk of death for every kilometer traveled on a motorcycle accident is 20 times higher than from a car crash [14]. The government of Lagos State, Southwest Nigeria, in 2012 passed a law banning the use of motorcycles on many major roads. The law prohibited motorcycle riders below 18 years and prescribed the use of standardized protective gear for all riders and passengers in the region. A survey done two years after the passage of this law saw a three-fold decrease in the proportion of death from motorcycle crashes in the region [15]. This current study found that 25.5% of road injuries involved motorcycles while explaining for 10% of all injuries that presented to the study site. The high prevalence in this current study, corroborated by other reports implicates motorcycle injuries to be a rising public health problem in Ghana, that requires urgent measures, education, and law enforcement to reduce associated morbidity.

Our finding of high prevalence of injuries from falls also calls for targeted policies, to focus especially on the extremes of age, reining in interventions such as making playgrounds safer, the use of safety gates, grab bars, improving general geriatric care and screening programs for elderly Ghanaians.

The pattern of leading causes of injuries in this study was similar to reports in Zambia Tanzania [7], Nigeria and Sierra-Leone, where the most common etiology was road traffic accidents, being responsible for 55% of injuries, followed by falls (17%) and then assaults (14%) [16].

There was a two-peak pattern of patient attendance to the Casualty and accident centre. These were around January/December, and April. These could probably have been accounted for by the national festive seasons that characterize these periods, namely the Christmas/New Year festivities, and Easter festivities respectively. Also, patient presentation generally peaked on Saturdays, which again, is consistent with the many socio-cultural weekly events such as weddings, funerals and outdooring ceremonies which characterize most weekends in many parts of the country. People would typically commute further distances to attend such events, thereby increasing the risk of road traffic accidents. This finding also favourably compares with that of other studies [17].

Our study, being retrospective was limited in the number and depth of variables considered. Inefficiencies in record keeping systems were also a limitation to data retrieval, evident by the quantity of unspecified information for some of the variables. This study did not record prehospital deaths, thus potentially skewing the mode of injuries that contributed most to mortality. Caution must also be exercised in extrapolating the results of this study to the general population as the single source of data may be a threat to external validity. This study, however, using a major hospital in the country as a case study, provides useful information on the pattern of injury occurrences and presentations which could inform health policy, as well as the generate hypotheses for future studies.

## Conclusion

Injury management is crucial to lowering the burden of morbidity and mortality in Ghana. While a more detailed audit of the casualty and accident management in the hospitals may provide in-depth knowledge on other aspects of the epidemiology of injuries, the trauma system in Ghana seems to leave much to be desired. The health system as a whole in Ghana needs to prioritize injury management with regards to surveillance, prevention, pre-hospital care, human resource, record keeping and access to care.

## Data Availability

Data and materials may be requested by contacting the corresponding author, Dr. Joachim Amoako.

## Abbreviations

LMIC: Lower-middle Income countries
RTA: Road traffic accident
SDG: Sustainable Development Goals
WHO: World Health Organization
